# Probing the clinical and brain structural boundaries of bipolar and major depressive disorder

**DOI:** 10.1038/s41398-020-01169-7

**Published:** 2021-01-14

**Authors:** Tao Yang, Sophia Frangou, Raymond W. Lam, Jia Huang, Yousong Su, Guoqing Zhao, Ruizhi Mao, Na Zhu, Rubai Zhou, Xiao Lin, Weiping Xia, Xing Wang, Yun Wang, Daihui Peng, Zuowei Wang, Lakshmi N. Yatham, Jun Chen, Yiru Fang

**Affiliations:** 1 Clinical Research Center & Division of Mood Disorders, Shanghai Mental Health Center, Shanghai Jiao Tong University School of Medicine, Shanghai, China; 2 Department of Psychiatry, University of British Columbia, Vancouver, Canada; 3 Department of Psychiatry, Icahn School of Medicine at Mount Sinai, New York, USA; 4 Department of Psychology, Provincial Hospital Affiliated to Shandong University, Jinan, China; 5 Shanghai Pudong New District Mental Health Center, Shanghai, China; 6 Department of Medical Psychology, Xinhua Hospital, Shanghai Jiao Tong University School of Medicine, Shanghai, China; 7 Division of Mood Disorders, Shanghai Hongkou District Mental Health Center, Shanghai, China; 8 CAS Center for Excellence in Brain Science and Intelligence Technology, Shanghai, China; 9 Shanghai Key Laboratory of Psychotic Disorders, Shanghai, China

**Keywords:** Bipolar disorder, Diagnostic markers, Depression

## Abstract

Bipolar disorder (BD) and major depressive disorder (MDD) have both common and distinct clinical features, that pose both conceptual challenges in terms of their diagnostic boundaries and practical difficulties in optimizing treatment. Multivariate machine learning techniques offer new avenues for exploring these boundaries based on clinical neuroanatomical features. Brain structural data were obtained at 3 T from a sample of 90 patients with BD, 189 patients with MDD, and 162 healthy individuals. We applied sparse partial least squares discriminant analysis (s-PLS-DA) to identify clinical and brain structural features that may discriminate between the two clinical groups, and heterogeneity through discriminative analysis (HYDRA) to detect patient subgroups with reference to healthy individuals. Two clinical dimensions differentiated BD from MDD (area under the curve: 0.76, *P* < 0.001); one dimension emphasized disease severity as well as irritability, agitation, anxiety and flight of ideas and the other emphasized mostly elevated mood. Brain structural features could not distinguish between the two disorders. HYDRA classified patients in two clusters that differed in global and regional cortical thickness, the distribution proportion of BD and MDD and positive family history of psychiatric disorders. Clinical features remain the most reliable discriminant attributed of BD and MDD depression. The brain structural findings suggests that biological partitions of patients with mood disorders are likely to lead to the identification of subgroups, that transcend current diagnostic divisions into BD and MDD and are more likely to be aligned with underlying genetic variation. These results set the foundation for future studies to enhance our understanding of brain–behavior relationships in mood disorders.

## Introduction

Mood disorders, primarily major depressive disorder (MDD) and bipolar disorder (BD), jointly affect up 20% of the population over their lifetime^1^ and rank amongst the most significant causes of disability worldwide^1^. Both disorders present with recurrent depressive psychopathology while manic symptoms are the diagnostic hallmark of BD^2^. BD is further subdivided in two main subtypes; type I (BD-I), characterized by episodes of mania, and type II (BD-II), that presents with hypomanic episodes^2^.

Regardless of subtype, the course of BD is dominated by depressive symptoms^3,4^. In about two-thirds of cases, BD begins with depression^5^ while the first manic episode does not emerge for another 5–9 years on average^6^. The significant symptomatic overlap between MDD and BD across the entire course of these disorders and the dominance of depressive symptoms at the early stages of BD often delays the correct identification of BD cases^7^ with significant implications for treatment decisions^8^, clinical outcomes^9^, and service use^10^.

Accordingly, the differentiation of BD from MDD depression is an issue of ongoing research interest. Initial investigations focused exclusively on the patients’ symptom profiles and found that concomitant depressive and manic symptoms, higher number of overall episodes and early age at the onset of depression (<25 years) were more characteristic of BD than MDD^11–15^. The advent of neuroimaging offered the opportunity to examine whether brain measures could assist in discriminating between MDD and BD depression. Direct comparisons using mass-univariate analyses identified a wide array of brain structural and functional differences but with marked inter-study variability^16,17^. More recently the field has turned towards machine learning techniques which use multivariate algorithms to parse clinical samples into more biologically homogeneous groups. The largest study to date applied a support vector machine to volumetric data from 596 depressed patients with MDD, 158 depressed patients with BD and 777 healthy individuals recruited at four different sites^18^. The results were not reproducible across sites but the features the influenced intersite variation were not systematically examined. Nevertheless, the volume of the middle, superior and inferior frontal gyrus emerged as the most promising discriminatory features in each site.

Collectively the available evidence suggests that differentiating MDD and BD depression using either clinical symptoms or brain structural data remains a challenge. In response, the aim of the current study was to test whether a data-driven approach enhanced by the application of advanced multivariate methods may improve the discrimination of depressed patients with MDD or BD and the identification of the most distinguishing features. To achieve this, we capitalized on a large sample of patients and healthy participants (total sample *n* = 441) who were assessed and scanned at a single site. First, we used sparse partial least squares discriminant analysis (s-PLS-DA)^19^ to test whether we could detect clinical or brain structural measures that could reliably differentiate the two patient groups. This supervised machine learning method is an extension of the classic PLS; it enables categorical classification with a specific focus on feature selection^20^, and has been used successfully in neuroimaging^21^. Second, we tested whether we could identify subgroups of patients in reference to healthy individuals based on brain structural data. We used heterogeneity through discriminative analysis (HYDRA)^22^, a novel semi-supervised classifier that can detect multiple subgroups, if present, within the clinical sample, and then further examine whether such brain-based clusters might differ in terms of clinical features.

## Methods

### Sample

The pooled analysis sample comprised 90 patients with BD, 189 patients with MDD and 162 healthy individuals, all of Han Chinese origin (Table 1, Supplementary Table S1 and Supplementary Figs. S1, S2). All study procedures were undertaken at the Division of Mood Disorders, Shanghai Mental Health Center (SMHC), Shanghai Jiao Tong University School of Medicine, China, and are described in detail in the Supplementary Material. Briefly, after providing written informed consent, all participants, regardless of diagnosis, were screened to exclude medical and neurological comorbidity, substance and alcohol problem use and contraindications to MRI. All patients fulfilled criteria for either MDD or BD as defined in the fourth edition of the Diagnostic and Statistical Manual of Mental Disorders (DSM-IV)^23^, while the healthy comparison individuals had no personal or family history of psychiatric disorders. All patients were recruited for participation in two clinical trials (Clinical Trail Registry ID: NCT01938859 and NCT01764867). However, all the data used here were acquired at enrollment and prior to study treatment initiation. Patients were required to meet the following criteria for a depressive episode: (a) total score ≥17 in the 17-item Hamilton Depression Rating Scale (HAMD)^24^ and a score of ≥2 in the HAMD item for depressed mood and, (b) total score ≤10 in the Young Mania Rating Scale (YMRS)^25^. Patients with rapid cycling, mixed affective episodes, other psychiatric comorbidity and patients who received electroconvulsive or other neuromodulation treatments were excluded.

**Table 1:** Sample characteristics.

Variable	Patients with BD *N* = 90	Patients with MDD *N* = 189	Healthy Individuals *N* = 162
Age (years)	26.44 (5.87)^a,b^	27.84 (6.19)	27.77 (5.21)
Sex (Male/Female)	36/54	71/118	67/95
Education (years)	14.78 (2.43)^a^	14.98 (2.83)	15.51 (2.75)
Marital status			
Single	57 (69.51)^a,b^	105 (58.01)^a^	102 (62.96)
Married/cohabitation	18 (21.95)	67 (37.02)	60 (37.04)
Divorce/separation	7 (8.54)	9 (4.97)	0 (0.00)
Employment status			
Unemployment	13 (16.46)^a,b^	52 (32.30)^a^	9 (5.56)
Part-time employment	3 (3.80)	8 (4.97)	2 (1.23)
Full-time employment	38 (48.10)	73 (45.34)	91 (56.17)
Students	25 (31.65)	28 (17.39)	60 (37.04)
BMI	22.67 (3.89)	21.76 (3.62)	22.42 (2.92)
HAMD total score	21.82 (4.48)^a^	21.20 (4.11)^a^	1.58 (1.76)
YMRS total score	2.46 (3.17)^a,b^	1.11 (1.51)	1.00 (1.92)
HAMA total score	17.70 (7.04)	17.05 (6.44)	n/a
Age of onset (years)	20.16 (5.52)^b^	25.13 (7.17)	n/a
Number of episodes	5.37 (6.31)^b^	1.65 (1.08)	n/a
Illness duration (months)	73.48 (57.31)^b^	45.00 (55.30)	n/a
GAF score	51.62 (6.39)^b^	54.67 (6.50)	n/a
SDS score			n/a
SDS-work/study	6.68 (2.86)	7.37 (8.96)	
SDS-social life	6.48 (2.70)	6.35 (2.53)	
SDS-family life	6.27 (2.72)	5.73 (2.81)	
Positive family history of psychiatric disorders	16 (21.05)	26 (15.95)	0 (0.00)
Never medicated	52 (67.53)	114 (70.80)	n/a

Continuous variables are shown as mean (standard deviation); Employment status, marital status, medication status, and family history are shown as number (percentage).

*BD* bipolar disorder, *MDD* major depressive disorder, *BMI* body mass index, *HAMD* Hamilton depression scale, YMRS Young Mania rating scale, *HAMA* Hamilton anxiety scale, *GAF* global assessment of function, *SDS* Sheehan disability scale, *n/a* not available or not applicable.

^a^Differences between each diagnostic group and healthy individuals at *P*_FDR_ < 0.05.

^b^Differences between the two diagnostic groups at *P*_FDR_ < 0.05; further details in the Supplemental Material.

### Clinical assessment

The diagnostic assignment of patients was based on consensus by specialist psychiatrists according to the DSM-IV criteria for each mood disorder. In all participants, information was collected on sociodemographic variables, psychopathology was rated with the 17-item-HAMD and the YMRS, an estimate of executive function was obtained using a computerized version of the Wisconsin Card sorting test (WCST)^26^, and body mass index (BMI)^27^ was used as a global estimate of metabolic status. In patients only, further assessments were undertaken using the Hamilton anxiety scale (HAMA)^28^, the global assessment of functioning (GAF)^29^ and the Sheehan disability scale (SDS)^30^. Information on age of onset, duration of illness, number of episodes, and medication status was also collected.

### Neuroimaging

In all participants, T_1_-weighted magnetic resonance imaging (MRI) data were acquired on the same Siemens Magnetom Verio 3 T scanner (Erlangen, Germany) at the Radiological Department of the SMHC. Details of the data acquisition and processing are provided in the Supplement. Following preprocessing, the gray matter was parcellated and segmented into distinct regions, using the FreeSurfer 6.0 software suite (http://surfer.nmr.mgh.harvard.edu/), yielding 68 cortical thickness measures, 16 subcortical volume measures and 16 hippocampal subfield volumes (Supplementary Table S2).

### Statistical analyses

#### Conventional group comparisons

Conventional univariate analyses were undertaken to compare patients to healthy individuals and conduct comparisons amongst the clinical subgroups in terms of clinical, cognitive, and brain structural data (Supplemental Material).

#### s-PLS-DA

We chose s-PLS-DA to identify features discriminating between MDD and BD patients as this approach accommodates correlated variables while the penalty prevents overfitting and enables robust feature selection^19^. We constructed two datasets, one comprising the brain structural variables (Supplementary Table S2) and another comprising sociodemographic measures (age, sex, years of education, marital status), clinical course specifiers (number of episodes, illness duration, age at onset), medication use, family history, BMI, cognitive measures from the WCST, and item scores from each clinical instrument (Table 1). Patients with BD were considered together regardless of subtype. The variables of each dataset were used as predictors while the clinical diagnoses (i.e., MDD and BD) were modeled as outcomes. s-PLS-DA was implemented using the mixOmics package (version 6.8.0)^31^. The detailed code is available on the website (https://github.com/AmirhosseinModabbernia/DepressionStudy). Each dataset was treated as a separate block, with the option for follow-up with multiblock analyses^32^. For each block, we created an optimization sample (80% of the original) and a held-out sample (20% of the original) by random sampling. We tuned the s-PLS-DA parameters based on the classification error rate with respect to the number of selected variables, for one component at a time, up to a maximum of 5. Optimization further involved cross validation (folds = 5) repeated ten times in the optimization sample. Following optimization, the best model was identified by calculating the area under the receiver operating characteristic (AUROC) in the held-out sample. Statistical significance was based on permutation, whereby we randomly permuted the optimization sample 10,000 times. For each permuted sample, we performed s-PLS-DA using the optimal parameters identified in the original model and retested the weights identified in the permuted data on the held-out set. To test generalizability, we followed an established multi-holdout procedure^33^, whereby we created ten randomly selected optimization/held-out samples using the 80/20 split and repeated the above analyses. Of the ten samples, the one yielding the lowest *P*-value in the held-out set was chosen as the best model based on a Bonferroni corrected *P* < 0.05.

#### HYDRA

This is a non-linear machine learning algorithm for integrated binary classification and subpopulation clustering^22^. Key advantages of HYDRA, over other machine learning techniques, are that it disposes of the need for a priori specification of the number of clusters and does not use similarity measures for clustering as such measures are susceptible to the effect of non-specific factors such as age and sex. The detailed code can be found on https://github.com/evarol/HYDRA. Classification in HYDRA is based on indices of deviation between a clinical and the healthy reference group; healthy individuals are separated from the clinical sample using a convex polytope formed by combining multiple linear hyperplanes. This confers an additional advantage to HYDRA because the multiple hyperplanes model potential heterogeneity within clinical samples while their combination extends linear max-margin classifiers to the non-linear space. The regional cortical thickness measures and subcortical volumes (Supplementary Table S2) of all patients and healthy individual and entered into the algorithm as input features and sex and age were modeled as covariates. The clinical diagnoses of MDD and BD were not entered as variables to enable data-driven classification independent of clinical categorization; therefore the patients’ data were considered together without reference to diagnostic labels. We used 5-fold cross validation to determine the clustering stability for a cluster range of 2–5. The resultant clustering solutions were evaluated using the adjusted Rand index (ARI), adjusted for the chance grouping of elements; the solution with the highest ARI was chosen. The generalizability of the clustering solutions was tested through permutation (Supplementary Material). Subgroups identified as above were compared in terms of their demographics, clinical, and cognitive characteristics.

## Results

### Sample characteristics

Patients with BD were marginally younger and had fewer years of education compared to healthy individuals; regardless of diagnosis, a higher number of patients were unemployed (Table 1). Clinical differences between MDD and BD groups were noted in the total YMRS and GAF scores, age of onset and illness duration and total number of episodes (Table 1). Regardless of diagnosis, patients underperformed in the WCST compared to healthy individuals in terms of percentage of perseverative responses, percentage of total errors, percentage of conceptual level responses, number of categories completed, and total number required for completing the first category (Supplementary Table S3).

Of the 90 patients with BD, 31 had BD-Type I (BD-I) and 59 had BD-Type II (BD-II). Compared to patients with BD-II, patients with BD-I had higher HAMD and HAMA total scores (Supplementary Table S4). No differences were noted between bipolar subtypes in cognitive characteristics (Supplementary Table S5).

### Differentiating between MDD and BD with s-PLS-DA

**Fig. 1:**
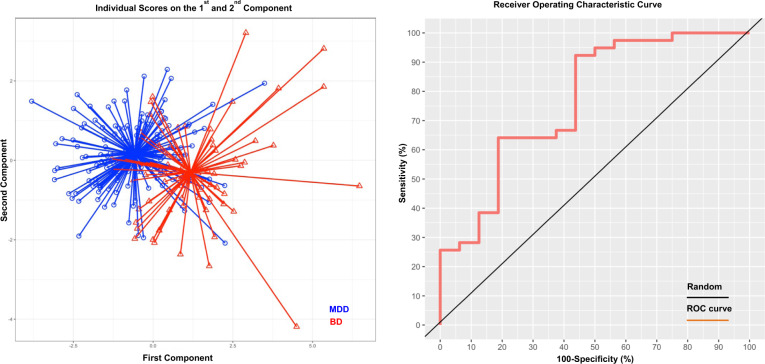
Sparse partial least squares discriminant analysis (s-PLS-DA) of clinical features differentiating bipolar disorder (BD) from major depressive disorder (MDD). Left: Two components differentiated BD from MDD. The star plot shows their centroid. The individual patient score on these components are presented as lines radiating from the centroid. Lines representing patients with MDD are shown in blue and lines representing patients with BD are shown in red. The variable weights for each component are presented in Supplementary Table S6. Right: The receiver operating characteristic curve derived from the s-PLS-DA differentiating BD from MDD based on the test samples; area under the curve (AUC) = 0.73, *P* = 0.001 (additional details in Supplementary Fig. S3A, B).

The s-PLS-DA differentiated between MDD and BD based on clinical (AUROC = 0.76, *P* = 0.001) but not brain structural features (AUROC = 0.63, *P* = 0.47). (Fig. 1 and Supplementary Fig. S3A, B) and identified two components that distinguished between BD and MDD (Fig. 1). The loading weights of each feature in each component are shown in Supplementary Table S6. In the first component, the highest loadings were observed for measures of disease severity/chronicity (i.e., age of onset, total number of episodes, and illness duration), and for clinical symptoms of irritability/aggression, increased activity/energy, flight of ideas, and psychic and somatic anxiety. In the second component, the highest loading concerned elevated mood and somatic symptoms of anxiety. Age, sex, and medication status had zero or minimal loadings in both components.

### Neuroanatomical clustering of patients with HYDRA

**Fig. 2:**
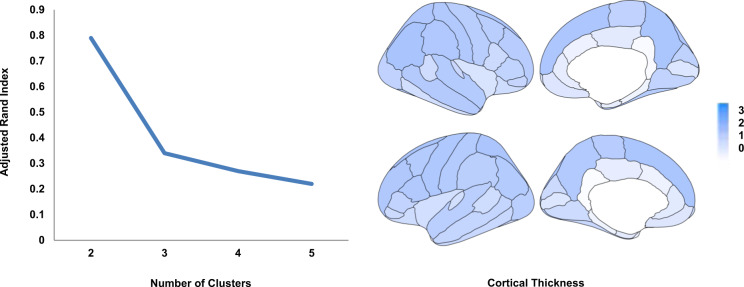
Patient clusters derived through heterogeneity through discriminative analysis (HYDRA). Left: HYDRA identified two clusters of patients based on the adjusted Rand index (ARI). This is shown as a clear peak in ARI values for the 2-cluster solution. Right: The two clusters differed in global and regional cortical thickness; the Cohen’s *d* of these regional differences is mapped onto the corresponding cortical regions (additional details in Supplemental Material).

Cluster stability in HYDRA showed a significant peak in ARI values favoring the 2-cluster solution (Fig. 2). Cluster 1 comprised 151 patients, of whom 93 (61.6%) had a diagnosis of MDD, 17 (11.3%) had a diagnosis of BD-I and 41 (27.1%) had a diagnosis of BD-II. Cluster 2 comprised 128 patients, of whom 96 (75%) had a diagnosis of MDD, 14 (10.9%) had a diagnosis of BD-I and 18 (14.1%) had a diagnosis of BD-II. A nearly equal proportion of patients with BD-I were assigned to each cluster. However, there were more patients with BD-II and less patients with MDD in cluster 1 than those in cluster 2 (*χ*^2^ = 7.46, df = 2, *P* = 0.024). Details of the cluster comparisons in demographic and clinical features are presented in Supplementary Table S7. Notably, there were no differences in age, sex, age of onset, illness duration, and medication status (all *P* > 0.20). The only clinical feature that was statistically significant different between the two clusters was the percentage of patients with positive family history for psychiatric disorders, which was higher in cluster 1 than that cluster 2 (*P*_FDR_ = 0.03). Additionally, cluster 1 had lower cortical thickness globally (right *P* = 8.33E−43, left *P* = 8.84E−46) and in all cortical regions (range of *P*-values: 6.07E−30 to 6.03E−05) (Fig. 2B and Supplementary Fig. S4) although less pronounced along the cingulate cortex (Fig. 2). No significant differences were noted for subcortical volumes (range of *P*-values: 0.009 to 0.81).

### Group-level analyses of brain structural data

Conventional group-level analyses comparing patients with MDD or BD to healthy individuals identified widespread differences in cortical thickness but not in subcortical volumes (Supplemental Material and Supplementary Figs. S5–S7). Patients with BD had reduced cortical thickness in the left caudal middle frontal gyrus and the left pars opercularis compared to patients with MDD (*P*_FDR_ < 0.05). All other pairwise comparisons did not yield significant results (Supplemental Material). In univariate correlation analyses, only the SDS-study score was negatively correlated with the cortical thickness of the left temporal pole in patients with BD (Supplementary Fig. S8).

## Discussion

We applied multivariate methods to clinical and brain structural data from depressed patients with BD or MDD. The results showed that the two disorders in our study could be distinguished from each other with moderate accuracy based on clinical features but not brain structure. Clustering patients using healthy individuals as a “normative” reference identified two clusters that crossed diagnostic boundaries, and highlighted cortical thickness as a brain structural feature most likely to differentiate patients irrespective of symptoms.

Consistent with prior studies^11–15^, we confirmed the value of clinical features in discriminating between MDD and BD. The performance of the discriminant analysis presented here is comparable to that of Inoue et al.^14^ and Leopacher et al.^15^, who used conceptually similar analyses to ours on Japanese and US samples, and reported AUC ranging between 0.74 and 0.84. Data-driven feature selection implemented in s-PLS-DA identified of two clinical dimensions (i.e., components) underpinning the differentiation of the two disorders. The main dimension (component 1), included clinical features indicative of irritability, agitation, anxiety, and flight of ideas. The second component involved elevated mood and physical symptoms of anxiety. The irritability/agitation dimension, in the context of a depressive episode, dates back to the concept of “agitated depression”, first proposed by Weygandt^34^, as the combination of depressed mood, psychomotor agitation, and flight of ideas. The concept was revived and revised as “anxious agitated depression”^35^, while further research has focused on whether or not elevated mood should be considered part of “agitated depression” or assigned to a new construct of “mixed states”^36,37^. The present results suggest that irritability/agitation/anxiety dimension is the most discrepant dimension between BD and MDD depression and that mood elevation is a secondary and separate dimension. To some extent, these findings align with genetic findings indicating the mood elevation may have distinct genetic underpinnings from depression, either in the context of BD or MDD^38,39^.

Brain structural data did not differentiate patients with BD from patients with MDD either when using s-PLS-DA or conventional group-level analyses. These findings are aligned with a recent meta-analysis of group-level comparisons of BD and MDD which found evidence of a continuum of brain structural abnormalities, with deficits being generally more pronounced in BD than MDD, with the exception of the cingulate cortical regions that seen similarly affected in both disorders^40^. A similar pattern emerges from multivariate comparisons of BD to MDD; although initial small studies appeared promising^41^, the largest and most reliable study to date by Matsuo et al.^18^ showed that such results are unlikely to generalize.

By contrast, each diagnostic group showed significant differences when compared to healthy individuals both in group-level and multivariate analyses. The group-level analyses largely recapitulate the findings of the large studies conducted by the BD and MDD working groups of the Enhancing Neuroimaging Genetics through Meta-Analysis (ENIGMA) Consortium^41–45^. We identified two patient clusters using heterogeneity through HYDRA, a semi-supervised machine learning algorithm that classifies patients in reference to healthy individuals. The two clusters transcended diagnostic boundaries and did not differ in demographic, clinical and cognitive features. However, cluster 1 differed from cluster 2, as it was associated with lower global and regional cortical thickness, the distribution of BD and MDD and higher positive family history for psychiatric disorders. These findings align with previous studies showing associations between brain structure in mood disorders and specific polymorphisms^46,47^, and a more recent large-scale study on in 51,665 individuals which showed that cortical thickness is influenced by multiple genetic polymorphisms some of which overlap with genetic risk loci for mood disorders^48^.

Several limitations of the current study should be noted. First, the cross-sectional nature of the available data precludes examination of the developmental trajectories of brain structural differences between patients and controls. Second, despite the careful assessment of the patients participating in this study, there is always a possibility that some MDD patients may present with manic or hypomanic episodes as a future date. Third, evidence from us^49^ and others^50^ suggests that brain structure can be influenced by multiple environmental factors, primarily quality of family life and exposure to significant psychosocial adversity; differential exposure of individuals in the current sample to such influences may account to the clusters identified. Fourth, the addition of genetic information to clustering models in future studies may lead to more fine-grained partitions of the clinical groups. Fifth, assessment of cognition in this sample was limited to a single test of abstraction and cognitive flexibility, thus precluding a more detailed examination of other cognitive features that may be closely related to structural changes. Sixth, patients in the current study were recruited for randomized clinical trials and cannot be considered representative of MDD and BD. Thus, the reproducibility of the findings reported here will require independent replication in larger and epidemiologically derived samples. Seventh, the current study focused exclusively on brain morphometry because brain structural data have the highest translational potential. It is possible that the inclusion of data from other modalities could lead to a different or a more refined partition of the clinical sample and this is worth pursuing in future studies.

In conclusion, our study provides support for the notion that agitated and mixed states being more characteristic of BD depression. The brain structural findings suggests that biological partitions of patients with mood disorders are likely to lead to the identification of subgroups, that transcend current diagnostic divisions into BD and MDD and are more likely to be aligned with underlying genetic variation.

## Supplementary information

Supplemental Material
