# Supplementary material for: Probing the clinical and brain structural boundaries of bipolar and major depressive disorder
Source: Transl Psychiatry. 2021 Jan 14;11:48. doi: 10.1038/s41398-020-01169-7 (PMC7809029; doi:10.1038/s41398-020-01169-7)
Supplement: Supplementary file 1 — Supplemental Material [file 41398_2020_1169_MOESM1_ESM.docx]

**Probing the clinical and brain structural boundaries of bipolar and major depressive disorder**

**Supplemental Material**

**1.Supplementary Methods**

- 1. **Samples**

Supplementary Figure S1. Flowchart of study sample selection

Supplementary Table S1. Comparison of patients who agreed or refused brain imaging

- 1. **Cognitive assessment**
  2. **Neuroimaging acquisition parameters**

**1.4** **MRI segmentation and quality assurance**

Supplementary Table S2. List of Freesurfer derived variable

Supplementary Figure S2. Analysis sample following quality control

**1.5** **Heterogeneity Through Discriminative Analysis (HYDRA)**

1.5.1 Permutation testing

**2.Supplementary Results**

**2.1 Medication status of patients**

**2.2 Comparison of cognitive task performance between MDD and BD**

Supplementary Table S3. Cognitive task performance in MDD and BD

**2.3 Comparison of sociodemographic and clinical features between bipolar subtypes**

Supplementary Table S4. Sociodemographic and clinical features in BD-I and BD-II

**2.4 Comparison of cognitive task performance between bipolar subtypes**

Supplementary Table S5. Cognitive task performance in BD-I and BD-II

**2.5 Sparse Partial Least Squares Discriminant Analyses (s-PLS-DA)**

Supplementary Table S6. Loading weights of the socio-clinical features differentiating

Bipolar disorder from major depressive disorder in the s-PLS-DA

Supplementary Figure S3A. Receiver operating characteristics curve in the optimization

sample differentiating BD from MDD in terms of clinical features

Supplementary Figure S3B. Receiver operating characteristics curve in the optimization

Sample differentiating BD from MDD in terms of neuroimaging features

**2.6 HYDRA**

Supplementary Figure S4. Mean cortical thickness differences in the HYDRA-derived

Clusters

Supplementary Table S7. Comparisons of patients in the HYDRA-derived Clusters

**2.7 Neuroimaing: Group-level analyses**

**2.7.1 Cortical thickness**

2.7.1.1. Bipolar Disorder versus Healthy Individuals

2.7.1.2 Major Depressive Disorder versus Healthy Individuals

2.7.1.3 Bipolar Disorder versus Major Depressive DIsorder

Supplementary Figure S5. Effect size of case-control differences in regional cortical thickness in MDD and BD

**2.7.2 Subcortical volumes**

2.7.2.1 Bipolar Disorder versus Healthy Individuals

2.7.2.2 Major Depressive Disorder versus Healthy Individuals

2.7.2.3 Bipolar Disorder versus Major Depressive Disorder

Supplementary Figure S6. Effect size of case-control differences in regional subcortical volumes in MDD and BD

**2.7.3 Hippocampal subfields**

2.7.3.1 Bipolar Disorder versus Healthy Individuals

2.7.3.2 Major Depressive Disorder versus Healthy Individuals

2.7.3.3 Bipolar Disorder versus Major Depressive Disorder

Supplementary Figure S7. Effect size of case-control differences in the volume of the hippocampal subfield in MDD and BD

**2.6 Univariate correlations between brain structural measures and clinical and cognitive variables**

Supplementary Figure S8. Univariate correlations between each clinical, cognitive variables and social function variables and mean regional cortical thickness and subcortical volume patients with BD or MDD

**2.7 Comparison in neuroimaging features between bipolar subtypes**

**2.8 Comparision between each BD subtype to healthy individuals and patients with MDD**

2.8.1 Comparison of cognitive task performance between each BD subtype to healthy individuals and patients with MDD

2.8.2 Comparison of cortical thickness, subcortical and hippocampal subfield volumes between each BD subtype to healthy individuals and patients with MDD

**1. Supplementary Methods**

**1.1 Samples**

Patients with bipolar disorder (BD) or major depressive disorder (MDD) were recruited from the Division of Mood Disorders, Shanghai Mental Health Center (SMHC), Shanghai Jiao Tong University School of Medicine via clinician referrels from July 2013 to June 2017 in order to participate into two clinical trials (http://www.clinicaltrials.gov; Clinical Trial Registry ID: NCT01938859 and NCT01764867). The studies were approved by the Institutional Review Board of the SMHC and were conducted in accordance with the Declaration of Helsinki. All participants received a detailed explanation of the study and provided the written informed consent at enrolment.

All the patients were screened by well-trained psychiatry residents with the Chinese version of the MINI-International Neuropsychiatric Interview (M.I.N.I.) (1). Psychopathology was rated using the Hamilton Depression Scale-17 (HAMD) (2) and the Young Mania Rating Scale (YMRS) (3). The final diagnoses were made by two senior psychiatrists according to the Diagnostic and Statistical Manual for Mental Disorders-Fourth Edition-Text Revision (DSM-IV-TR). Patients fulfilled the following inclusion criteria: (a) age 18-45 years; (b) of Han Chinese origin; (c) able to give consent; (d) in a current depressive episode defined as having a total HAMD score of 17 or above and scoring 2 or above in the HAMD item 1 (depressed mood) and having a total YMRS score of 10 or less; (d) minimum 8 years of education (i.e. junior high school level or above); (e) right-handed based on self-report.

Patients were excluded if they had (a): rapid cycling bipolar disorder; (b) current mixed affective episode; (c) clinical psychiatric comorbidity in the past 6 months; (d) comorbid personality disorder; (e) substance or alcohol abuse in the past 6 months; (f) any history of electroconvulsive therapy or repetitive transcranial magnetic stimulation; (g) any past or current significant medical condition or head trauma; (h) any contraindications for MRI scan (e.g. metal implant or claustrophobia). For women only, we excluded those pregnant or planning to get pregnant or lactating.

During the same period, healthy individuals were recruited from the local community and college students through a social medial application. Eligibility was based on the following criteria (a) age 18-45 years; (b) of Han Chinese origin; (c) no lifetime personal history of mental disorders or substance use based on the MINI; (d) HAMD<7 and YMRS<5 on the day of their assessment; (e) no family history of major psychiatric disorders; (f) minimum 8 years of education (i.e. junior high school level or above); (g) right-handed based on self-report; (h) any past or current medical condition or head trauma; (i) any contraindications for MRI scan (e.g. metal implant or claustrophobia). For women only, we excluded those pregnant or planning to get pregnant or lactating.

After screening, a total of 738 participants (219 BD, 321 MDD, and 196 HCs) were enrolled. The demographic information of all participants and the clinical characteristics of patients were collected using a study-specific form. Patients had additional clinical assessments using the Hamilton Anxiety Scale (HAMA) (4), the Global Assessment of Function (GAF) (5) and the Sheehan Disability Scale (SDS) (6). The information about psychotropic medication prior to study enrolment was also collected and coded in terms of name and class of the agents, daily dose, efficacy, and side effects. Additional clinical information concerned age of onset, duration of illness, number of episodes, and family history of psychiatric disorders.

Following enrolment and prior to being randomized into the clinical trial, patients were asked to undertake magnetic resonance imaging. Of the clinical sample enrolled, 105 patients with BD, 225 patients with MDD had an MRI scan while the remaining patients refused. All the healthy individuals recruited agreed to be scanned (n=196). We will refer to the group with MRI data as the “study sample”. The sample selection process is shown in Supplementary Figure S1. Comparisons of the demographic and key clinical characteristics of the patients who agreed or refused to be scanned are summarized in Supplementary Table S1. Compared to those that refused, patients with BD who agreed to MRI were younger and were less likely to be married and unemployed. They had a higher age of onset and were more likely to be medication naïve. Compared to those that refused, patients with MDD who agreed to MRI were more likely to be single and had a lower HAMD score. The differences in patients with BD but not in patients with MDD survived FDR correction.

**Supplementary Figure S1. Flow-chart of study sample selection**

**
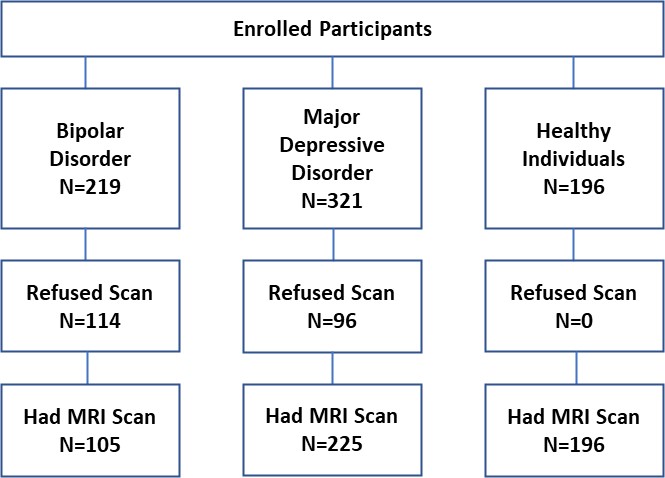
**

| **Supplementary Table S1. Comparison of patients who agreed or refused neuroimaging** | | | | |
| --- | --- | --- | --- | --- |
| **Variable** | **Patients with BD who agreed to MRI** | **Patients with BD who refused MRI** | **Patients with MDD who agreed to MRI** | **Patients with MDD who refused MRI** |
| Age (years) | 27.23 (7.16)^1,3^ | 31.52 (11.72) | 28.66 (7.70) | 28.97 (6.39) |
| Sex (Male/Female) | 46/59 | 51/64 | 86/139 | 41/55 |
| Education (years) | 14.78 (2.57) | 14.52 (3.52) | 14.85 (2.90) | 15.23 (2.80) |
| Marital status  Single  Married/cohabitation  Divorce/separation | 68 (69.39)^1,3^  23 (23.47)  7 (7.14) | 63 (58.88)  41(38.32)  3 (2.80) | 118 (55.66)^2^  84 (39.62)  10 (4.72) | 49 (52.13)  45 (47.87)  0 (0.00) |
| Employment state  Unemployment  Part-time employment  Full-time employment  Students | 17(17.89)^1,3^  3 (3.16)  47 (49.47)  28 (29.47) | 32 (30.48)  0 (0.00)  52 (49.52)  21 (20.00) | 62 (32.63)  8 (4.21)  84 (44.21)  36 (18.95) | 27 (31.40)  1 (1.16)  46 (53.49)  12 (13.95) |
| Bipolar Disorder, Type II | 62.00 (71.26) | 65.00 (65.00) | Not applicable | Not Applicable |
| HAMD total score | 21.62 (4.48) | 21.63 (4.38) | 21.14 (4.17)^2^ | 22.47 (4.14) |
| YMRS total score | 2.27 (3.04) | 2.20( 2.86) | 1.22 (1.68) | 1.22 (1.86) |
| HAMA total score | 17.22 (7.12) | 17.01 (6.71) | 17.12 (6.31) | 17.74 (6.69) |
| Age of onset (years) | 24.49 (9.69)^1,3^ | 20.42 (5.94) | 25.32 (8.13) | 25.62(7.17) |
| Number of episodes | 5.38 (6.02) | 5.93 (7.23) | 1.63 (1.04) | 1.75 (1.65) |
| Illness duration (months) | 81.48 (62.41) | 92.39 (91.01) | 42.17 (57.85) | 38.12 (65.92) |
| GAF score | 51.81(6.18) | 54.14 (7.83) | 54.69 (7.77) | 55.37 (7.07) |
| SDS score  SDS-study/work  SDS-social life  SDS-family life | 6.82 (2.66)  6.51 (2.54)  6.15 (2.62) | 6.42 (2.81)  6.31 (2.61)  5.98 (2.76) | 7.24 (8.32)  6.34 (2.45)  5.59 (2.78) | 6.62 (2.23)  6.13 (2.36)  5.70 (2.74) |
| Positive family history of psychiatric disorders | 25 (26.88) | 25 (24.51) | 33 (17.46) | 20 (21.28) |
| Never medicated | 58 (63.04)^1,3^ | 47 (49.19) | 134 (71.28) | 60 (69.77) |
| Alcohol abuse | 0 (0.00) | 3 (2.91) | 0 (0.00) | 2 (2.15) |
| Drug abuse | 0 (0.00) | 0(0.00) | 0 (0.00) | 1 (1.08) |
| Continuous variables are shown as mean (standard deviation); BD subtype, medication status, family history and substance abuse are shown as number (percentage). BD = bipolar disorder; MDD = major depressive disorder; HAMD = Hamilton Depression Scale; YMRS = Young Mania Rating Scale; HAMA = Hamilton Anxiety Scale; GAF = Global Assessment of Function; SDS = Sheehan Disability Scale; ^1^ differences between the scanned versus not-scanned patients with BD at p<0.05 uncorrected; ^2^ differences between the scanned versus not-scanned patients with MDD at p<0.05 uncorrected; ^3^ differences between the scanned versus not-scanned patients with BD at p<0.05 following FDR correction; There were not differences between the scanned and the not-scanned patients with MDD at FDR p<0.05. | | | | |

**1.2 Cognitive assessment**

The cognitive function of all participants was evaluated on the same day they had their scan using the Chinese computerized version of the Wisconsin Card Sorting Test (WCST). The WCST assesses “attention, visual processing, abstract concept formation, and cognitive flexibility (7). Task performance was assessed based on the percentage of errors (PE), the percentage of perseverative responses (PPR), the percentage of perseverative errors (PPE), the percentage of conceptual level responses (PCLR), the number of categories completed (CC) and the total number of trials required for completing the first category (TCFC).

**1.3 Neuroimaging acquisition parameters**

All imaging data were acquired on the same Siemens Magnetom Verio 3T scanner (Erlangen, Germany) at the Radiological Department of the SMHC. T1-weighted structural imaging data were acquired using a 3D magnetization-prepared rapid gradient-echo (MPRAGE) sequence using the following parameters: repetition time (TR) = 2530ms; time to echo (TE) = 3.65ms; inversion time (TI) = 1100ms; field of view (FOV) = 256×256mm; matrix = 256×256; 1 mm isotropic voxels; flip angle (FA) = 7º; bandwidth = 180 Hz/pixel, echo spacing = 8.5ms; in-plane acceleration factor = 2.

**1.4 MRI segmentation and quality assurance**

Neuroimaging data processing was implemented in FreeSurfer 6.0 (http://surfer.nmr.mgh.harvard.edu/). The steps included removal of non-brain tissue using a hybrid watershed/surface deformation procedure (8), automated Talairach transformation, segmentation of the subcortical white matter and deep gray matter volumetric structures (9, 10), intensity normalization (11), tessellation of the boundary between the gray and white matter, automated topology correction (12, 13), and surface deformation following intensity gradients to optimally place the gray/white matter boundaries and gray/cerebrospinal fluid borders at the location where the greatest shift in intensity defines the transition to the other tissue class. After the subcortical segmentation, the automated algorithm developed by Iglesias and colleagues (14) was applied to segment the hippocampal subfields. These processes yielded 68 cortical thickness, 16 subcortical volume measures, and 16 hippocampal subfield volumes (Supplementary Table S2). The volumes of subcortical regions and hippocampal subfields were adjusted by the intracranial volume (ICV) via a formula: adjusted volume = raw volume - *b**(ICV - mean ICV). The detailed information was described elsewhere (15).

| **Supplementary Table S2. Definition of the imaging variables** | |
| --- | --- |
| **Cortical Thickness** | |
| Banks of the superior temporal sulcus | Derived from cortical reconstruction using Freesurfer v6.0; left and right measures were considered separately |
| Caudal anterior cingulate cortex | Derived from cortical reconstruction using Freesurfer v6.0; left and right measures were considered separately |
| Caudal middle frontal gyrus | Derived from cortical reconstruction using Freesurfer v6.0; left and right measures were considered separately |
| Cuneus | Derived from cortical reconstruction using Freesurfer v6.0; left and right measures were considered separately |
| Entorhinal cortex | Derived from cortical reconstruction using Freesurfer v6.0; left and right measures were considered separately |
| Fusiform gyrus | Derived from cortical reconstruction using Freesurfer v6.0; left and right measures were considered separately |
| Inferior parietal lobule | Derived from cortical reconstruction using Freesurfer v6.0.; left and right measures were considered separately |
| Inferior temporal gyrus | Derived from cortical reconstruction using Freesurfer v6.0.; left and right measures were considered separately |
| Isthmus cingulate cortex | Derived from cortical reconstruction using Freesurfer v6.0.; left and right measures were considered separately |
| Lateral occipital gyrus | Derived from cortical reconstruction using Freesurfer v6.0; left and right measures were considered separately |
| Lateral orbitofrontal gyrus | Derived from cortical reconstruction using Freesurfer v6.0; left and right measures were considered separately |
| Lingual gyrus | Derived from cortical reconstruction using Freesurfer v6.0; left and right measures were considered separately |
| Medial orbitofrontal gyrus | Derived from cortical reconstruction using Freesurfer v6.0; left and right measures were considered separately |
| Middle temporal gyrus | Derived from cortical reconstruction using Freesurfer v6.0; left and right measures were considered separately |
| Parahippocampal gyrus | Derived from cortical reconstruction using Freesurfer v6.0; left and right measures were considered separately |
| Paracentral gyrus | Derived from cortical reconstruction using Freesurfer v6.0; left and right measures were considered separately |
| Pars opercularis | Derived from cortical reconstruction using Freesurfer v6.0; left and right measures were considered separately |
| Pars orbitalis | Derived from cortical reconstruction using Freesurfer v6.0; left and right measures were considered separately |
| Pars triangularis | Derived from cortical reconstruction using Freesurfer v6.0; left and right measures were considered separately |
| Pericalcarine gyrus | Derived from cortical reconstruction using Freesurfer v6.0; left and right measures were considered separately |
| Postcentral gyrus | Derived from cortical reconstruction using Freesurfer v6.0; left and right measures were considered separately |
| Posterior cingulate cortex | Derived from cortical reconstruction using Freesurfer v6.0; left and right measures were considered separately |
| Precentral gyrus | Derived from cortical reconstruction using Freesurfer v6.0; left and right measures were considered separately |
| Precuneus | Derived from cortical reconstruction using Freesurfer v6.0; left and right measures were considered separately |
| Rostral anterior cingulate cortex | Derived from cortical reconstruction using Freesurfer v6.0; left and right measures were considered separately |
| Rostral middle frontal gyrus | Derived from cortical reconstruction using Freesurfer v6.0; left and right measures were considered separately |
| Superior frontal gyrus | Derived from cortical reconstruction using Freesurfer v6.0; left and right measures were considered separately |
| Superior parietal lobule | Derived from cortical reconstruction using Freesurfer v6.0; left and right measures were considered separately |
| Superior temporal gyrus | Derived from cortical reconstruction using Freesurfer v6.0.; left and right measures were considered separately |
| Supramarginal gyrus | Derived from cortical reconstruction using Freesurfer v6.0; left and right measures were considered separately |
| Frontal pole | Derived from cortical reconstruction using Freesurfer v6.0; left and right measures were considered separately |
| Temporal pole | Derived from cortical reconstruction using Freesurfer v6.0; left and right measures were considered separately |
| Transverse temporal gyrus | Derived from cortical reconstruction using Freesurfer v6.0; left and right measures were considered separately |
| Insula | Derived from cortical reconstruction using Freesurfer v6.0; left and right measures were considered separately |
| **Subcortical Volumes** | |
| Thalamus | Derived from segmentation using Freesurfer v6.0; left and right measures were considered separately following ICV correction |
| Hippocampus | Derived from segmentation using Freesurfer v6.0; left and right measures were considered separately following ICV correction |
| Hippocampal subfields  Hippocampal tail  Subiculum (Sub)  Presubiculum (Presub)  Granule cell layer (GCL)  Molecular layer (ML)  Cornu ammonis 1 (CA1)  Cornu ammonis 2/3 (CA2/3)  Cornu ammonis 4 (CA4) | Freesurfer v6.0; left and right measures were considered separately following ICV correction |
| Caudate nucleus | Derived from segmentation using Freesurfer v6.0; left and right measures were considered separately following ICV correction |
| Nucleus Accumbens | Derived from segmentation using Freesurfer v6.0; left and right measures were considered separately following ICV correction |
| Pallidum | Derived from segmentation using Freesurfer v6.0; left and right measures were considered separately following ICV correction |
| Putamen | Derived from segmentation using Freesurfer v6.0; left and right measures were considered separately following ICV correction |
| Amygdala | Derived from segmentation using Freesurfer v6.0; left and right measures were considered separately following ICV correction |
| Lateral ventricles | Derived from segmentation using Freesurfer v6.0; left and right measures were considered separately following ICV correction |

Quality control for each structural dataset was implemented first by visual inspection by a trained neuroradiologist and then according to the publically available protocol from the ENIGMA initiative (http://enigma.ini.usc.edu/) and using Qoala-T (16). Following quality assessment, 85 individuals were excluded as shown in Supplemental Figure S2. The final analysis sample comprised 441 individuals with good quality structural MRI data.


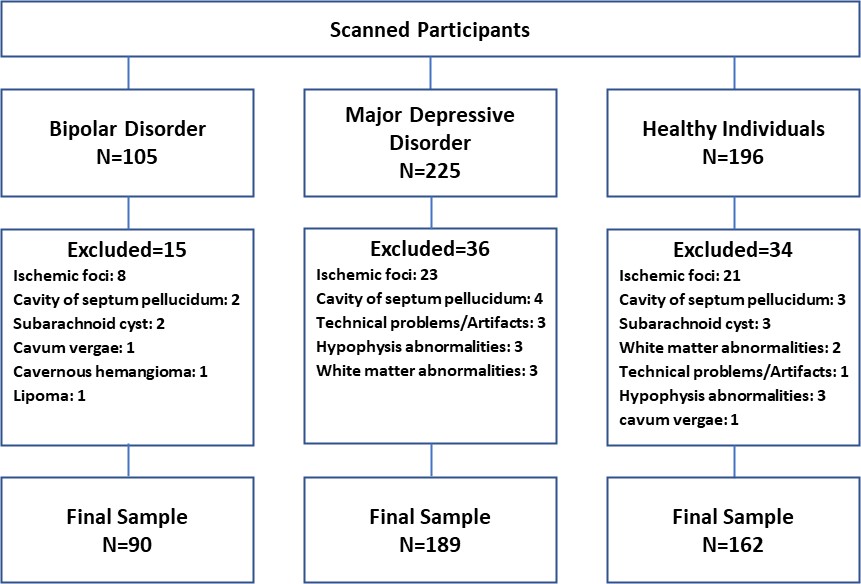


**Supplementary Figure S2: Flow chart for analysis sample selection**

- 1. **HYDRA**
     1. **Permutation testing**

We followed the procedure described by Kaczkurkin et al (17). First, participants were randomly divided into two equal groups (n=81 each); one group was considered a “control group” and the other was considered a “clinical group”. These samples were permuted 100 times in HYDRA to derive a null distribution of the results that would be free of any disorder-related influences. Second, we repeated the same analyses (with 100 permutations) but this time the clinical group included data from the actual patients to derive a real patient distribution. Clustering stability was always assessed using the adjusted Rand index (ARI). The stability of the clusters derived from the null distribution was compared to that of the actual patients.

**2. Supplementary Results**

**2.1 Medication status of the patients**

Of the 90 patients with BD in the analysis sample, data on medication status were available for 77; of these, only 17 (22.08%) were prescribed medication at the time of enrolment. Specifically,10 patients (12.99%) were on antipsychotics, 4 patients (5.19%) were on antidepressants, 4 (5.19%) patients were on antiepiletics (valproate=3; lamotrigine=1), 1 patient (1.30%) was on lithium, 2 patients (2.60%) were on sedative-hypnotics, and 1 patient (1.30%) on a Chinese medicine (Shu Gan Jie Yu capsule). There were no differences between the medicated and never medicated patients with BD in terms of age, sex, bipolar subtype, age of onset, number of episodes, duration of illness, total scores of the HAMD, YMRS, HAMA, GAF, and SDS.

Of the 186 patients with MDD in the analysis sample, data on medication status were available for 160; of these, 38 patients (23.75%) were prescribed medication at the time of enrolment. Specifically, 31 patients (19.38%) were on antidepressants, 7 (4.38%) patients were on antipsychotics, 2 (1.25%) patients were on antiepileptics (valproate), 8 patients (5.00%) were on sedative-hypnotics, and 2 patients (1.25%) were on a Chinese medicine (Shu Gan Jie Yu capsule). Compared to never medicated patients with MDD, those prescribed medications were mostly male, had more episodes, and higher HAMA total score but lower SDS and YMRS total scores (FDR p<0.05).

**2.2 Comparison of cognitive task performance between MDD and BD**

Data from the WCST were available for 73 patients with BD, 159 patients with MDD and 144 healthy individuals. The means and standard deviation for all WCST output measures are shown in Supplementary Table S3. Compared to healthy individuals, patients with BD underperformed in terms of perseverative errors, perseverative responses, conceptual level responses, categories completed, and total number required for completing the first category. Compared to healthy individuals, patients with MDD underperformed in terms of errors (total and perseverative), perseverative responses, conceptual level responses, categories completed, and total number required for completing the first category. These differences survived FDR correction. Differences between the two patient groups did not survive correction for multiple testing.

| **Supplementary Table S3. Cognitive task performance in MDD and BD** | | | |
| --- | --- | --- | --- |
| **Task Performance Variable** | **Patients with BD**  **N=73** | **Patients with MDD**  **N=159** | **Healthy Individuals**  **N=144** |
| WCST, PE | 31.15 (16.56) ^1,3^ | 33.95 (19.40)^1,3^ | 25.07 (11.10) |
| WCST, PPR | 16.20 (13.65)^1,3^ | 19.86 (17.79)^1,3^ | 12.22 (9.07) |
| WCST, PPE | 4.70 (3.62) | 5.27 (5.67)^3^ | 4.70 (4.45) |
| WCST, PCLR | 65.33 (19.13)^1,3^ | 62.98 (21.59)^1,3^ | 72.91 (12.77) |
| WCST, CC | 5.29(1.47)^1,3^ | 5.08(1.70)^1,3^ | 5.78 (0.76) |
| WCST, TCFC | 18.75(15.22)^3^ | 18.85(13.75)^3^ | 16.76 (11.28) |
| All variables are shown as mean (standard deviation); 1 = case-control differences at uncorrected p<0.05; 2 = differences between the diagnostic groups at uncorrected p<0.05; 3 = case-control differences at FDR p<0.05; There were not differences between the diagnostic groups at FDR p<0.05; The comparisons between patient groups and healthy individuals were adjusted by age and education year; The comparisons between two patient groups were adjusted for age, years of education, HAMA total score, HAMD total score and YMRS total score. WCST = Wisconsin card sorting test; MDD = Major depressive disorder; BD = Bipolar disorder; PE = percentage of errors; PPR = percentage of perseverative responses; PPE = percentage of perseverative errors; PCLR = percentage of conceptual level responses; CC = number of categories completed; TCFC = total number required for completing the first category. | | | |

**2.3 Comparison of sociodemographic and clinical features between bipolar subtypes**

Of the 90 patients with BD in the analysis sample, 31 had BD-Type I (BD-I) and 59 had BD-Type II (BD-II). Compared to patients with BD-II, patients with BD-I had higher HAMD and HAMA total scores (Supplementary Table S4).

| **Supplementary Table S4. Sociodemographic and clinical features in BD-I and BD-II** | | |
| --- | --- | --- |
| **Variable** | **Patients with BD-I**  **N = 31** | **Patients with BD-II**  **N = 59** |
| Age (years) | 25.87 (6.50) | 26.74 (5.55) |
| Sex (Male/Female) | 10/21 | 26/33 |
| Education (years) | 14.23 (2.65) | 15.09 (2.26) |
| Marital status  Single  Married/cohabitation  Divorce/separation | 23 (82.14)  4 (14.29)  1 (3.57) | 35 (63.64)  14 (25.45)  6 (10.17) |
| Employment state  Unemployment  Part-time employment  Full-time employment  Students | 8 (30.77)  2 (7.67)  8 (30.77)  8 (30.77) | 8 (9.26)  1 (1.85)  31 (57.41)  17 (31.48) |
| BMI | 22.09 (4.37) | 22.90 (3.71) |
| HAMD total score | 23.52 (4.55)^1^ | 20.98 (4.24) |
| YMRS total score | 1.83 (2.06) | 2.75 (3.54) |
| HAMA total score | 20.92 (7.42)^1^ | 16.20 (6.40) |
| Age of onset (years) | 19.21 (6.28) | 20.58 (5.15) |
| Number of episodes | 4.90 (4.56) | 5.58 (7.01) |
| Illness duration (months) | 70.88 (63.29) | 74.66 (54.99) |
| GAF score | 52.36 (6.67) | 51.31 (6.38) |
| SDS score  SDS-study/work  SDS-social life  SDS-family life | 6.36 (3.11)  6.24 (2.79)  6.00 (2.93) | 7.10 (2.73)  6.60 (2.67)  6.40 (2.63) |
| Positive family history of psychiatric disorders | 4 (16.00) | 12 (23.53) |
| Never medicated | 15 (62.50) | 37 (71.15) |
| Continuous variables are shown as mean (standard deviation); Medication status and family history are shown number (percentage). BD=bipolar disorder; BMI=Body Mass Index; HAMD=Hamilton Depression Scale; YMRS=Young Mania Rating Scale; HAMA=Hamilton Anxiety Scale; GAF=Global Assessment of Function (GAF); SDS=Sheehan Disability Scale; ^1^differences between each diagnostic group at FDR p<0.05. | | |

**2.4 Comparison of cognitive task performance between bipolar subtypes**

Of the 90 patients with BD, complete data on the WCST were available in 73; of those 23 had BD-I and 48 had BD-II. No difference survived FDR correction between patients with BD-I or BD-II in any of the WCST variables.

| **Supplementary Table S5. Cognitive task performance in BD-I and BD-II** | | |
| --- | --- | --- |
| **Task Performance Variable** | **Patients with BD-I**  **N=23** | **Patients with BD-II**  **N=48** |
| WCST, PE | 35.26 (18.74) | 29.03 (15.07) |
| WCST, PPR | 20.22 (16.20) | 14.10 (11.76) |
| WCST, PPE | 5.55 (4.08) | 4.26 (3.31) |
| WCST, PCLR | 60.73 (21.42) | 67.72 (17.59) |
| WCST, CC | 5.20 (1.58) | 5.33 (1.42) |
| WCST, TCFC | 17.32 (14.15) | 19.50 (15.85) |
| All variables are shown as mean (standard deviation); 1 = differences at uncorrected p<0.05; 2 = differences at FDR p<0.05; The comparisons between two patient groups were adjusted for age, years of education, HAMA total score, HAMD total score and YMRS total score; WCST = Wisconsin card sorting test; PE = percentage of errors; PPR = percentage of perseverative responses; PPE = percentage of perseverative errors; PCLR = percentage of conceptual level responses; CC = number of categories completed; TCFC = total number required for completing the first category. | | |

**2.5 Spase Partlial Least Squares Discrinimant Analysis (s-PLS-DA)**

| **Supplementary Table S6. Loading weights of the socio-clinical features differentiating BD from MDD in the s-PLS-DA** | | | |
| --- | --- | --- | --- |
| **Component 1** | | **Component 2** | |
| **Variables** | **Weights** | **Variables** | **Weights** |
| Number of episodes | 0.393 | Respiratory symptoms (HAMA10) | 0.581 |
| Illness duration | 0.339 | Elevated mood (YMRS1) | 0.337 |
| Irritability (YMRS5) | 0.189 | Somatic symptoms (HAMA8) | 0.173 |
| Speech rate and amount (YMRS6) | 0.174 | Years of education | 0.157 |
| Somatic symptoms (HAMD13) | 0.172 | WCST-percentage of perseverative errors | 0.003 |
| Autonomic symptoms (HAMA13) | 0.164 | Increased Motor activity/energy (YMRS2) | 0 |
| Disruptive/aggressive behaviour (YMRS9) | 0.157 | Sexual Interest (YMRS3) | 0 |
| Elevated mood (YMRS1) | 0.148 | Sleep (YMRS4) | 0 |
| Anxiety-Psychic (HAMD10) | 0.144 | Irritability (YMRS5) | 0 |
| Somatic-Muscular (HAMA7) | 0.125 | Speech (rate and amount) (YMRS6) | 0 |
| Body mass index | 0.116 | Language/thought disorder (YMRS7) | 0 |
| Sexual interest (YMRS3) | 0.114 | Content (YMRS8) | 0 |
| Insomnia-Initial (HAMD4) | 0.112 | Disruptive/behaviour (YMRS9) | 0 |
| Increased Motor activity/energy (YMRS2) | 0.110 | Appearance (YMRS10) | 0 |
| Feelings of guilt (HAMD2) | 0.105 | Insight (YMRS11) | 0 |
| Agitation (HAMD9) | 0.105 | Depressed mood (HAMD1) |  |
| Language/thought disorder (YMRS7) | 0.099 | Feelings of guilt (HAMD2) | 0 |
| Appearance (YMRS10) | 0.096 | Insomnia-Initial (HAMD3) | 0 |
| Family history | 0.096 | Insomnia-Middle (HAMD5) | 0 |
| Hypochondriasis (HAMD15) | 0.094 | Insomnia-Delayed (HAMD6) | 0 |
| Insomnia-Middle (HAMD5) | 0.091 | Work and Interests (HAMD7) | 0 |
| Somatic-Sensory (HAMA8) | 0.088 | Retardation (HAMD8) | 0 |
| WCST-percentage of conceptual level responses | 0.084 | Agitation (HAMD9) | 0 |
| Weight loss (HAMD16) | 0.059 | Anxiety-Psychic (HAMD10) | 0 |
| Gastrointestinal symptoms (HAMA11) | 0.043 | Anxiety-Somatic (HAMD11) | 0 |
| Anxiety-Somatic (HAMD11) | 0.086 | Gastrointestinal symptoms (HAMD12) | 0 |
| Work and Interests (HAMD7) | 0.039 | Somatic symptoms-General (HAMD13) | 0 |
| Intellectual (HAMA5) | 0.036 | Genital symptoms (HAMD14) | 0 |
| Medication Status | 0.027 | Hypochondriasis (HAMD15) |  |
| Depressed mood (HAMD1) | 0.025 | Insight (HAMD17) | 0 |
| Content (YMRS8) | 0.024 | Anxious mood (HAMA1) |  |
| SDS-work/study | 0.022 | Tension (HAMA2) | 0 |
| Cardiovascular symptoms (HAMA9) | 0.014 | Fears (HAMA3) | 0 |
| Anxious mood (HAMA1) | 0.010 | Insomnia (HAMA4) |  |
| Sex | 0.007 | Intellectual (HAMA5) | 0 |
| Tension (HAMA2) | 0.002 | Depressed mood (HAMA6) | 0 |
| Insomnia (HAMA4) | 0 | Somatic-Muscular (HAMA7) | 0 |
| Genitourinary symptoms (HAMA12) | 0 | Cardiovascular symptoms (HAMA9) | 0 |
| Sleep (YMRS4) | 0 | Gastrointestinal symptoms (HAMA11) | 0 |
| SDS-family life | -0.004 | Genitourinary symptoms (HAMA12) | 0 |
| Suicide (HAMD3) | -0.006 | Autonomic symptoms (HAMA13) | 0 |
| Respiratory Symptoms (HAMA10) | -0.01 | Behavior at interview (HAMA14) | 0 |
| Insight (YMRS11) | -0.028 | SDS-work/study | 0 |
| SDS-social life | -0.028 | SDS-social life | 0 |
| Fears (HAMA3) | -0.031 | SDS-family life | 0 |
| Years of education | -0.035 | WCST-percentage of conceptual level responses | 0 |
| Behavior at interview (HAMA14) | -0.077 | WCST-percentage of errors | 0 |
| Depressed mood (HAMA6) | -0.099 | WCST-percentage of perseverative responses | 0 |
| WCST-percentage of perseverative errors | -0.109 | Sex | 0 |
| WCST-percentage of errors | -0.111 | Family History | 0 |
| Gastrointestinal symptoms (HAMD12) | -0.118 | Illness duration | 0 |
| Retardation (HAMD8) | -0.126 | Medication Status | 0 |
| Age | -0.130 | Age | 0 |
| Insight (HAMD17) | -0.146 | Body mass index | 0 |
| Genital symptoms (HAMD14) | -0.155 | Marital Status | 0 |
| WCST-percentage of perseverative responses | -0.158 | Age of onset | 0 |
| Marital Status | -0.176 | Insomnia-Initial (HAMD4) | -0.036 |
| Insomnia-Delayed (HAMD6) | -0.199 | Number of episodes | -0.254 |
| Age of onset | -0.377 | Weight loss (HAMD16) | -0.655 |
| The numbers next to the abbreviations of each scale correspond to the item numbers of that scale; BD=bipolar disorder; HAMD=Hamilton depression rating scale; HAMA=Hamilton anxiety scale; MDD=major depressive disorder; SDS=Sheehan Disability Scale; YMRS=Young mania rating scale; WCST=Wisconsin card sorting test | | | |

**
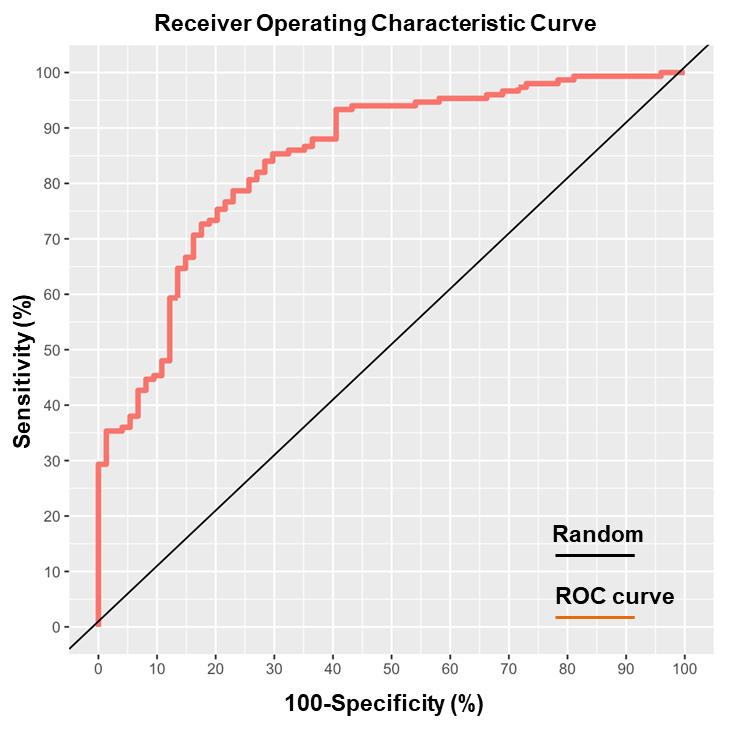
**

**Supplementary Figure S3A. Receiver operating characteristic (ROC) curve for clinical features differentiating bipolar disorder from major depressive disorder in the optimization set in the sparse spatial least squares analysis; area under the curve (AUC)=0.85; p=0.001**

**
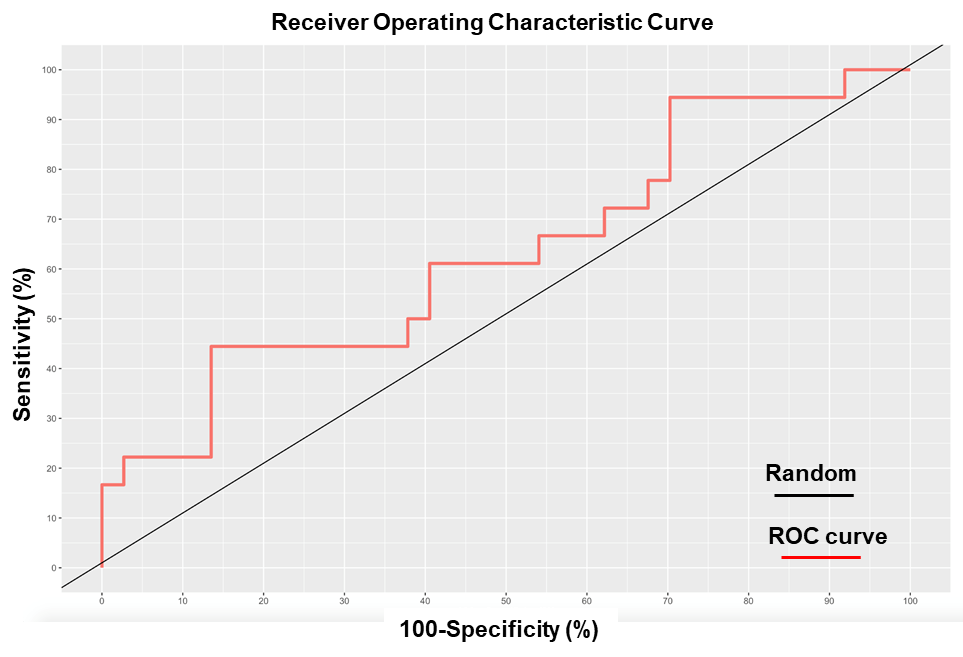
**

**Supplementary Figure S3B. Receiver operating characteristic (ROC) curve for neuroaimaging features differentiating bipolar disorder from major depressive disorder in the optimization set in the sparse spatial least squares analysis; area under the curve (AUC)=0.85, p= 0.40**

**2.6 HYDRA**

**
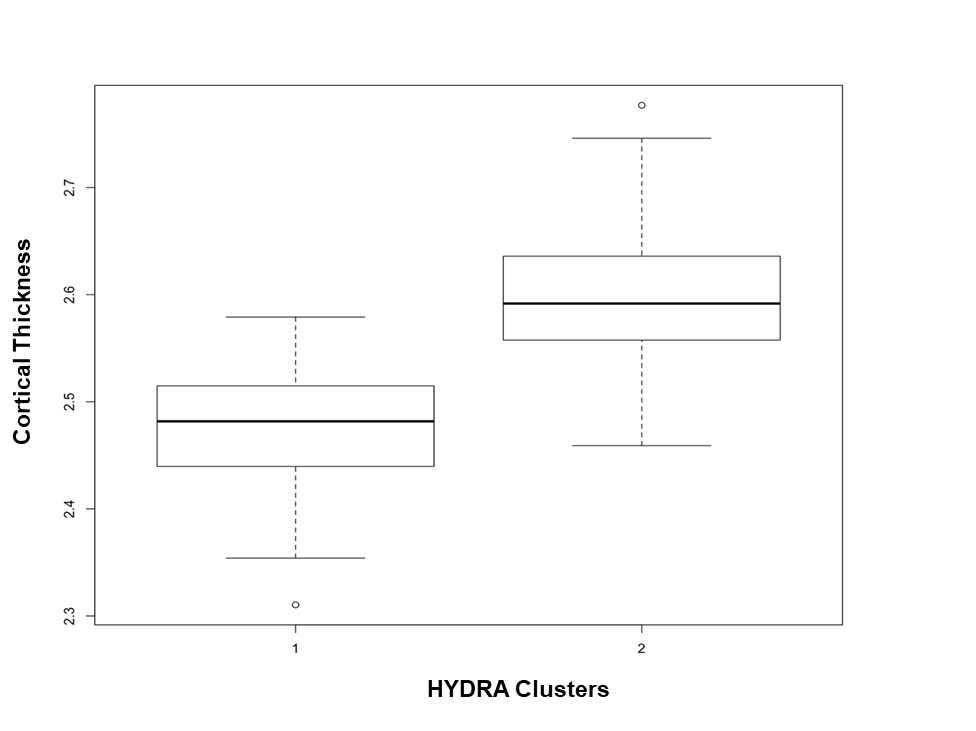
**The adjustment Rand Index (ARI) values for the cluser solutions were: 2-cluster:0.7627; 3-cluster:0.4029; 4-cluster: 0.2771; 5-cluster: 0.2271. The 2-cluster solution had the highest ARI in all the permuted samples with real patient data. The key difference between the two clusters was that cluster 2 had a higher mean cortical thickness (Supplementary Figure S4).

**Supplementary Figure S4. Mean cortical thickness difference in the two HYDRA-derived clusters.**

| **Supplementary Table S7.** **Comparisons of patients in the HYDRA-derived clusters** | | |
| --- | --- | --- |
| **Variable** | **Cluster 1**  **N=151** | **Cluster 2**  **N=128** |
| Age (years) | 27.16 (5.88) | 27.64 (6.40) |
| Sex (Male/Female) | 59/92 | 49/79 |
| Education (years) | 14.71 (2.89) | 15.15 (2.46) |
| Marital status  Single  Married/cohabitation  Divorce/separation | 86 (60.56)  46 (32.39)  10 (7.04) | 77 (63.11)  39 (31.97)  6 (4.92) |
| Employment state  Unemployment  Part-time employment  Full-time employment  Students | 35 (26.72)  4 (3.05)  63 (48.09)  29 (22.14) | 30 (27.27)  7 (6.36)  49 (44.55)  24 (21.82) |
| BMI | 21.65 (3.57) | 22.38 (3.83) |
| HAMD total score | 21.85 (4.02) | 21.18 (3.95) |
| YMRS total score | 1.59 (2.13) | 1.36 (2.13) |
| HAMA total score | 17.72 (6.75) | 16.70 (6.47) |
| Age of onset (years) | 23.04 (6.66) | 24.01 (7.48) |
| Number of episodes | 3.13 (4.76) | 2.18 (2.26) |
| Illness duration (months) | 55.00 (50.55) | 50.59 (59.6) |
| GAF score | 53.60 (6.47) | 54.66 (6.67) |
| SDS score  SDS-work/study  SDS-social life  SDS-family life | 6.81 (2.68)  6.46 (2.53)  5.93 (2.73) | 6.52 (2.71)  6.34 (2.67)  5.98 (2.85) |
| Positive family history of psychiatric disorders | 30 (23.62)^1,2^ | 12 (10.71) |
| Never medicated | 86 (66.15) | 81 (74.31) |
| Psychiatric comorbidity | 69 (49.64) | 46 (38.98) |
| WCST  PE  PPR  PPE  PCLR  CC  TCFC | 33.33 (18.85)  18.32 (16.74)  4.47 (4.54)^1^  63.36 (21.32)  5.13 (1.68)  19.35 (16.06) | 32.75 (18.28)  19.19 (16.62)  5.87 (5.68)  64.17 (20.31)  5.17 (1.57)  18.16 (11.49) |
| Continuous variables are shown as mean (standard deviation); Employment status, marital status, medication status, family history and psychiatric comorbidity are shown as number (percentage). BMI=Body Mass Index; HAMD=Hamilton Depression Scale; YMRS=Young Mania Rating Scale; HAMA=Hamilton Anxiety Scale; GAF=Global Assessment of Function; SDS=Sheehan Disability Scale; WCST = Wisconsin card sorting test; PE = percentage of errors; PPR = percentage of perseverative responses; PPE = percentage of perseverative errors; PCLR = percentage of conceptual level responses; CC = number of categories completed; TCFC = total number required for completing the first category; The comparisons of indexed in WCST between two patient groups were adjusted for age, years of education, HAMA total score, HAMD total score and YMRS total score; 1=differences between the two clusters P<0.05; 2=differences between the two clusters at P_FDR_<0.05; psychiatric comorbidity include general anxiety disorder, social anxiety disorder, panic disorder, agoraphobia, post-traumatic stress disorder, obsessive-compulsive disorder and eating disorders . | | |

**2.7 Neuroimaging: Conventional group-level analyses**

**2.7.1 Cortical thickness**

We compared regional cortical thickness measures between each patient group and healthy individuals by using ANCOVA and adjusted for age and sex; the statistical inference was set a p<0.05 following FDR correction. The effect size of case-control differences for each diagnosis expressed as Cohen’s *d*, is illustrated in Supplementary Figures S3a and S3b. The two clinical groups were also compared to each other using the same methodology and FDR correction for multiple testing.

**2.7.1.1 Bipolar disorder versus Healthy Individuals**

Compared to healthy individuals, patients with BD showed widespread reductions in cortical thickness. Regions with moderate Cohen’s d (Cohen’s d range: -0.30 to -0.35) that survived FDR correction at 0.05 included the bilateral caudal middle frontal gyrus, the bilateral rostral middle frontal gyrus, the bilateral superior parietal lobules, the left inferior parietal lobule, the left parahippocampal gyrus, the left paracentral gyrus, the left precuneus, and the left temporal pole. Further statistically significant reductions of smaller effect size (Cohen’s d range: -0.29 to -0.20) were noted in the bilateral pars trianularis, the bilateral posterior cingulate cortex the bilateral superior frontal gyrus, the bilateral superior temporal gyrus, the left bank of the superior temporal sulcus, the left pars opercularis, the left postcentral gyrus, the left supramarginal gyrus, the right fusiform gyrus, the right inferior parietal lobule, and the right lingual gyrus. By contrast, patients showed greater cortical thickness than healthy individuals in the left rostral anterior cingulate cortex, the left transverse temporal gyrus, the left cuneus, and the insula (Cohen’s d range from 0.09 to 0.10). These differences did not survive FDR correction.

**2.7.1.2 Major Depressive Disorder versus Healthy Individuals**

Compared to healthy individuals, patients with MDD also showed reductions in the cortical thickness of several brain regions, including the left lingual gyrus, the left inferior temporal gyrus, the left postcentral gyrus, the left rostral middle frontal gyrus, the left superior parietal lobule, the supramarginal gyrus, the right fusiform gyrus, and the right posterior cingulate cortex (FDR p<0.05; Cohen’s d range from -0.29 to -0.15). Additional but no reductions that just missed FDR corrected significance were noted in left pericalcarine and the right lingual gyrus (FDR p=0.063, 0.061; Cohen’s d -0.24 to -0.25). By contrast, patients with MDD showed greater cortical thickness than healthy individuals in the left frontal pole, the left isthmus cingulate cortex, the left cuneus and the right entorhinal cortex range from (Cohen’s d range from 0.09 to 0.29). Only the latter region was significant at FDR p<0.05.

**2.5.1.3 Bipolar Disorders versus Major depressive DIsorder**

Patients with BD had reduced cortical thickness in the left caudal middle frontal gyrus and the left pars opercularis compared to patients with MDD (FDR p<0.05; Cohen’s d range from -0.28 to -0.24).


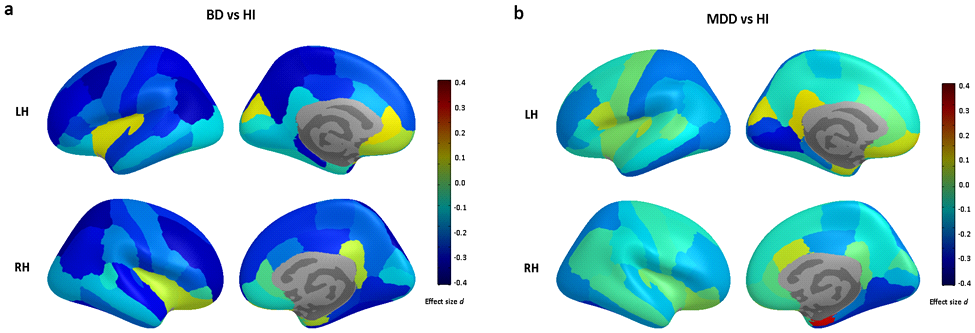


**Supplementary Figure S5. Effect size of case-control differences in cortical thickness.**  Panel A visualizes the Cohen’s d for the case-control differences in Bipolar Disorder (BD); Panel B visualizes the Cohen’s d for the case-control differences in Major Depressive Disorder (MDD); In both panels, warm colours indicate regions of higher cortical thickness in patients referenced to healthy individuals (HI) and cool colours regions of lower cortical thickness in patients referenced to healthy individuals.

**2.5.2 Subcortical volumes**

We compared regional subcortical volume measures between each diagnostic group and healthy individuals by using ANCOVA and adjusted for age, sex and TIV; the statistical inference was set a p<0.05 following FDR correction. The effect size of case-control differences for each case-control comparison expressed as Cohen’s d, is illustrated in Supplementary Figures S4a and S4b. The two clinical groups were also compared to each other using the same methodology and FDR correction for multiple testing.

**2.5.2.1 Bipolar Disorder versus Healthy Individuals**

Compared to healthy individuals, patients with bipolar disorder showed volumetric reductions in all subcortical regions (Cohen’s d range from -0.24 to -0.01) with the exception of the bilateral lateral ventricles (Cohen’s d range from 0.19 to 0.28) which were enlarged. None of these differences survived FDR correction.

**2.5.2.2 Major Depressive Disorder versus Healthy Individuals**

Compared to healthy individuals, patients with MDD showed volumetric reductions in most subcortical regions (Cohen’s d range from -0.18 to -0.005) but not in the bilateral hippocampus while the volumes of the bilateral caudate, the right putamen and the right lateral ventricles were enlarged (Cohen’s d range from 0.004 to 0.14). None of these differences survived FDR correction.

**2.5.2.3 Bipolar Disorder versus Major Depressive DIsorder**

There were no significant FDR corrected differences between patients with BD and patients with MDD in any subcortical volume (Cohen’s d range from -0.26 to 0.20).


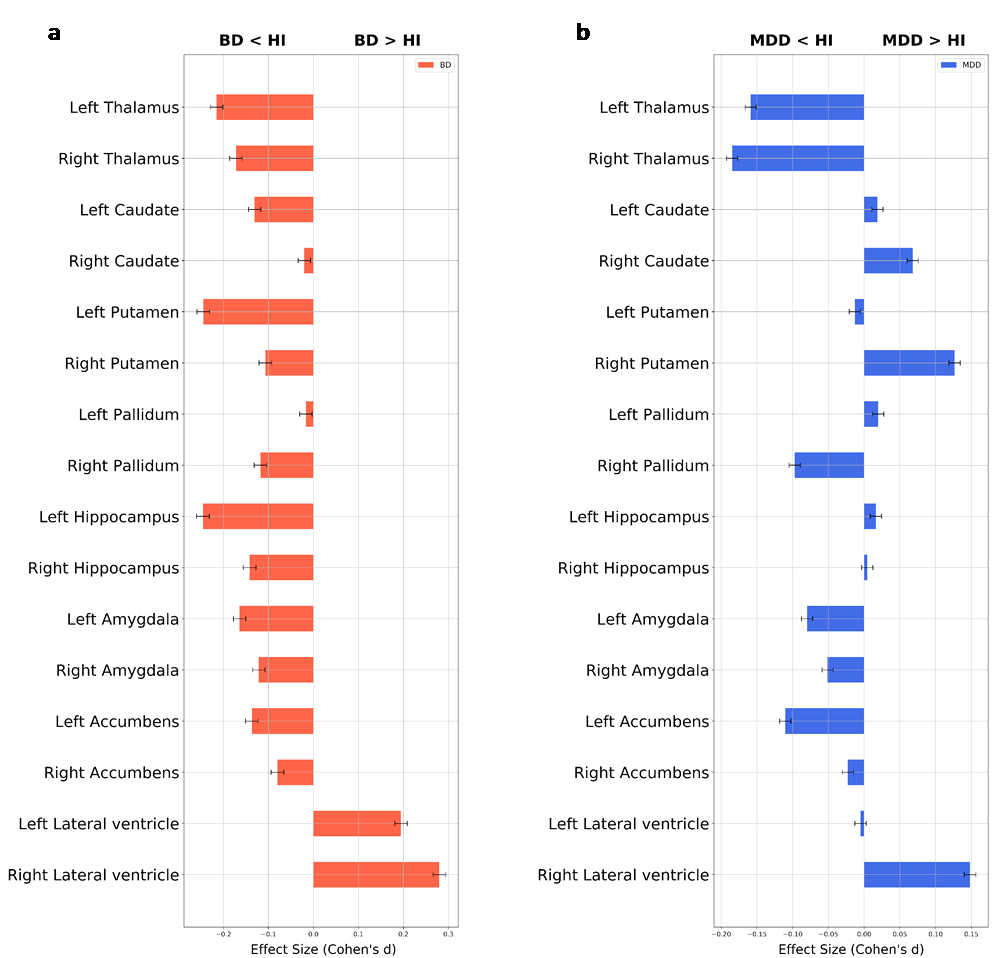


**Supplementary Figure S6. Effect size of case-control differences in subcortical volumes**. Panel A visualizes the Cohen’s d for the case-control differences in Bipolar Disorder (BD); Panel B visualizes the Cohen’s d for the case-control differences in Major Depressive Disorder (MDD)**.**

**2.5.3 Hippocampal subfields**

We compared the volumes of hippocampal subfields between each diagnostic group and healthy individuals by using ANCOVA and adjusted for age, sex and TIV; the statistical inference was set a p<0.05 following FDR correction. The effect size of case-control differences for each case-control comparison expressed as Cohen’s d, is illustrated in Supplementary Figures S5a and S5b. The two clinical groups were also compared to each other using the same methodology and FDR correction for multiple testing.

**2.5.3.1 Bipolar Disorder versus Healthy Individuals**

Compared to healthy individuals, patients with BD showed volumetric reductions in all hippocampal subfields (Cohen’s d range from -0.41 to -0.001) except for the right Cornu ammonis 3 (CA3, Cohen’s d=0.006) which was enlarged. None of these case-control differences survived FDR correction.

**2.5.3.3 Major Depressive Disorder versus Healthy Individuals**

Compared to healthy individuals, patients with MDD showed increased volumes in most hippocampal subfields but not in the CA1 while the volumes of the presubiculum, molecular layer, and CA3 were reduced (Cohen’s d range from -0.078 to -0.015). None of these case-control differences survived FDR correction.

**2.5.3.3 Bipolar Disorder versus Major Depressive DIsorder**

There were no significant FDR corrected differences between patients with BD and patients with MDD in any hippocampal subfield (Cohen’s d range from -0.30 to 0.04).


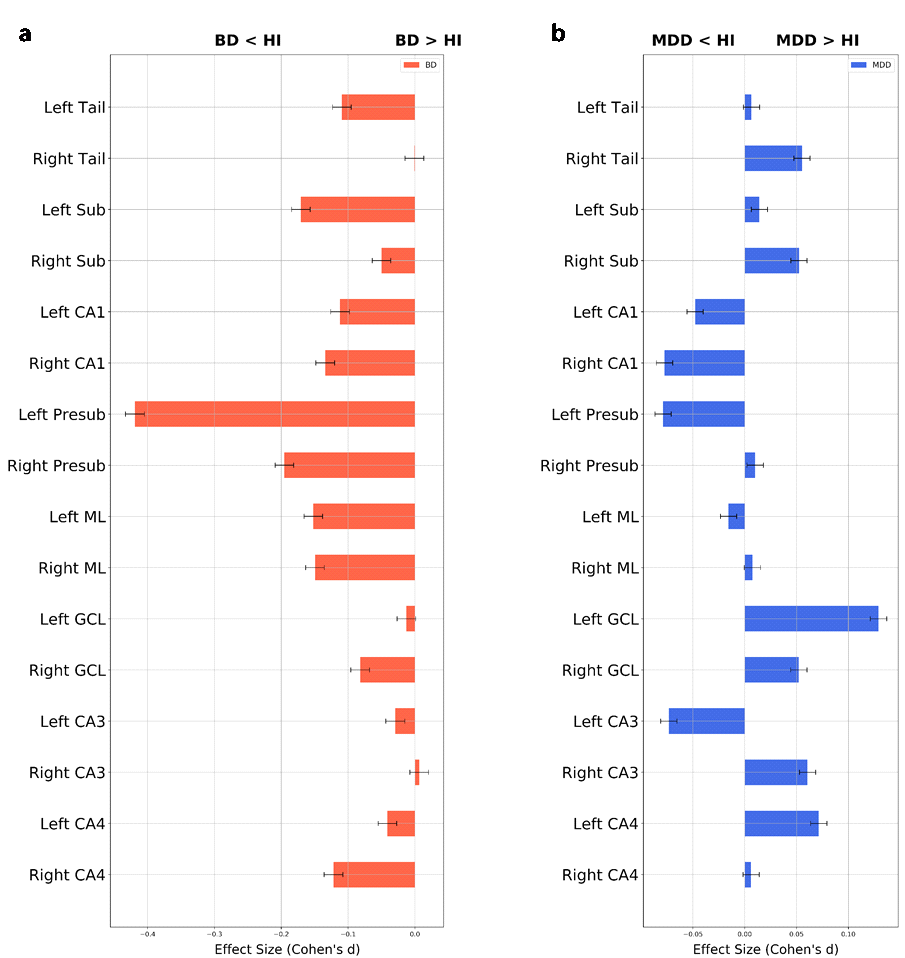


**Supplementary figure S7. Effect size of case-control differences in the volume of the hippocampal subfields.** Panel A visualizes the Cohen’s d for the case-control differences in Bipolar Disorder (BD); Panel B visualizes the Cohen’s d for the case-control differences in Major Depressive Disorder (MDD). Tail = Hippocampal tail; Sub = Subiculum; CA1 = Cornu ammonis 1; Presub = Presubiculum; ML = Molecular layer; GCL = Granule cell layer; CA3 = Cornu ammonis 3; CA4 = Cornu ammonis 4.

**2.6. Univariate correlations between brain structural measures and clinical and cognitive variables**

Separately within each patient group, we undertook univariate analyses correlating each brain structural measure with each clinical feature (i.e., illness duration, age of onset, number of episodes), current symptoms (i.e., HAMA, HAMD, YMRS), cognitive task performance measure (i.e., PE, PPR, PPE, PCLR), and social functioning (i.e. GAF, SDS-study, SDS-work, SDS-family).

In patients with BD, current symptoms, cognitive, and social functioning variables correlated with the cortical thickness in several brain regions. In addition, clinical features showed univariate correlations with some subcortical volumes as did social functioning variables and some hippocampal subfields volumes. Only the correlation between SDS-study and the cortical thickness of the left temporal pole survived FDR correction (r=-0.45, FDR p=0.01).

In patients with MDD, clinical features were mainly correlated with the cortical thickness of some brain regions. Current symptoms, the cognitive and social functioning were related to the cortical thickness of brain regions and the volumes of some hippocampal subfields. However, no correlation survived FDR.


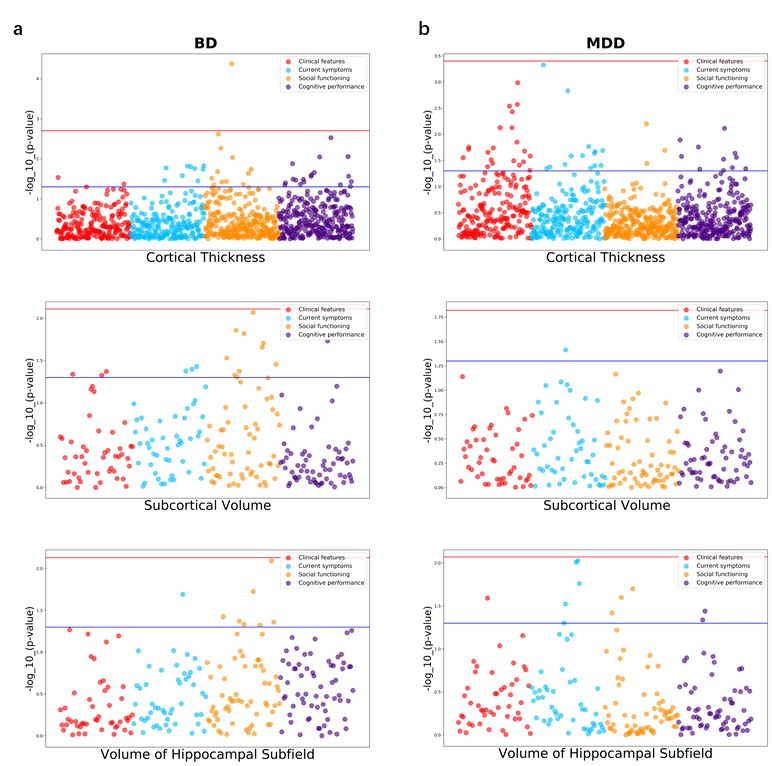


**Supplementary Figure S8. Univariate correlations between each clinical, cognitive, and social functioning and mean regional cortical thickness and subcortical volume patients with bipolar disorder (BD) or major depressive disorder (MDD).** Results are plotted as p-values from the corresponding correlations controlling for age and sex. The blue line shows the uncorrected threshold of statistical significance set at 0.05. The red line shows the threshold of statistical significance set at FDR p<0.05. Panel A visualizes the correlations in Bipolar Disorder (BD); Panel B visualizes the correlations in Major Depressive Disorder (MDD).

**2.7 Comparison in neuroimaging features between bipolar subtypes**

**Comparison of cortical thickness, subcortical and hippocampal subfield volumes in patients with BD-I versus BD-II**

Using the same methodology described for the main analyses, compared regional cortical and subcortical measures between bipolar subtypes. For each comparison, we report only results that survive p<0.05 following FDR correction for multiple testing. All the results reported below should be interpreted with caution because of the small sample size of patients with BD-I compared to those with BD-II.

Cortical thickness: No differences in regional cortical thickness between patients with BD-I and patients with BD-II survived FDR-correction (Cohen’s d range from -0.29 to 0.47).

Subcortical volumes: No differences in any subcortical volume between patients with BD-I and patients with BD-II survived FDR correction (Cohen’s d range from -0.34 to 0.36).

Hippocampal subfields: No differences in any hippocampal subfield volume between patients with BD-I and patients with BD-II survived FDR correction (Cohen’s d range from -0.38 to 0.17).

**2.8 Comparison between between each BD subtype to healthy individuals and patients with MDD**

**2.8.1 Comparison of cognitive task performance between each BD subtype to healthy individuals and patients with MDD**

We compared the cognitive performance of each subtype to healthy individuals and to patients with MDD. The comparisons between patients with BD subtypes and healthy individuals were adjusted for age and years of education. The analyses in patients with BD subtypes and patients with MDD were adjusted for age, years of education and the total scores of HAMA, HAMD and YMRS. We report only results that survive p<0.05 following FDR correction for multiple testing. Accordingly, patients with BD-I differed from healthy individuals in PE, PPR, PCLR and CC. Patients with BD-II differed from healthy individuals in PE, PCLR and CC. No differences were detected either between patients with BD-I and patients with MDD or between patients with BD-II and patients with MDD.

**2.8.2 Comparison of cortical thickness, subcortical and hippocampal subfield volumes between each BD subtype to healthy individuals and patients with MDD**

Using the same methodology described for the main analyses, compared regional cortical and subcortical measures between each bipolar subtype to healthy individuals and patients with MDD. For each comparison, we report only results that survive p<0.05 following FDR correction for multiple testing. All the results reported below should be interpreted with caution because of the small sample size of patients with BD-I compared to those with BD-II

**2.8.2.1 Comparison of each BD subtype to healthy individuals**

**Bipolar Disorder-I**

Cortical thickness: Following FDR-correction, patients with BD-I differed from healthy individuals only in the left posterior cingulate cortex and left precuneus (Cohen’s d -0.27 and -0.42).

Subcortical volumes: No differences between patients with BD-I and healthy individuals in subcortical volumes survived FDR-correction (Cohen’s d range from -0.35 to 0.49).

Hippocampal subfields: No differences between patients with BD-I and healthy individuals in hippocampal subfields volume survived FDR-correction (Cohen’s d range from -0.33 to -0.02).

**Bipolar Disorder-II**

Cortical thickness: Compared to healthy individuals, patients with BD-II showed reduction in cortical thickness in bilateral caudual middle frontal cortex, bilateral inferior parietal cortex, bilateral middle temporal gyrus, bilateral pars triangularis, bilateral central gyrus, bilateral posterior cingulate cortex, bilateral precentral gyrus, bilateral precuneus, bilateral rostral middle frontal gyrus, bilateral superior frontal gyrus, bilateral parietal cortex, bilateral superior temporal gyrus, left banks superior temporal sulcus, left parahippocampal gyrus, left paracentral gyrus, left pars opercularis, left supramarginal gyrus, left temporal pole, right fusiform, right lingual gyrus, right frontal pole and right transverse temporal gyrus (Cohen’s d range from -0.48 to -0.24).

Subcortical volumes: No differences between patients with BD-II and healthy individuals in subcortical volumes survived FDR correction (Cohen’s d range from -0.23 to 0.18).

Hippocampal subfields: No differences between patients with BD-II and healthy individuals in hippocampal subfields volume survived FDR correction (Cohen’s d range from -0.47 to 0.12).

**2.8.2.3 Comparison of each bipolar subtype to MDD**

**Bipolar Disorder-I**

Cortical thickness: No differences between patients with BD-I and patients with MDD in cortical thickness survived FDR correction (Cohen’s d range from -0.38 to 0.32).

Subcortical volumes: No differences between patients with BD-I and patients with MDD in subcortical volume survived FDR correction (Cohen’s d range from -0.38 to 0.35).

Hippocampal subfields: No differences between patients with BD-I and patients with MDD in hippocampal subfields volume survived FDR correction (Cohen’s d range from -0.40 to -0.07).

**Bipolar Disorder-II**

Cortical thickness: No differences between patients with BD-II and patients with MDD in cortical thickness survived FDR correction (Cohen’s d range from -0.44 to 0.14).

Subcortical volumes: No differences between patients with BD-II and patients with MDD in subcortical volume survived FDR correction (Cohen’s d range from -0.27 to 0.19).

Hippocampal subfields: No differences between patients with BD-I and patients with MDD in hippocampal subfields volume survived FDR correction (Cohen’s d range from -0.36 to 0.14).

**References**

1. Si, T. M., et al. Evaluation of the reliability and validity of Chinese version of the Mini-International Neuropsychiatric Interview in patients with mental disorders. *Chinese Mental Health Journal* **23**, 493-503 (2009).

2. Zheng, Y. P., et al. Validity and reliability of the Chinese Hamilton Depression Rating Scale. *Br J Psychiatry* **152**, 660-664 (1988).

3. Young, R. C., Biggs, J. T., Ziegler, V. E. & Meyer, D. A. A rating scale for mania: reliability, validity and sensitivity. *Br J Psychiatry* **133**, 429-435 (1978).

4. Hamilton, M. The assessment of anxiety states by rating. *Br J Med Psychol* **32**, 50-55 (1959).

5. Jones, S. H., Thornicroft, G., Coffey, M. & Dunn, G. A brief mental health outcome scale-reliability and validity of the Global Assessment of Functioning (GAF). *Br J Psychiatry* **166**, 654-659 (1995).

6. Leu, S. H., et al. Validity and reliability of the Chinese version of the Sheehan Disability Scale (SDS-C). *Asia Pac Psychiatry* **7**, 215-222 (2015).

7. Bryan, J.& Luszcz, M. A. Measurement of executive function: considerations for detecting adult age differences. *J Clin Exp Neuropsychol* **22**, 40-55 (2000).

8. Segonne, F., et al. A hybrid approach to the skull stripping problem in MRI. *Neuroimage* **22**, 1060-75 (2004).

9. Fischl, B., et al. Whole brain segmentation: automated labeling of neuroanatomical structures in the human brain. *Neuron* **33**, 341-55 (2002).

10. Fischl, B., et al. Sequence-independent segmentation of magnetic resonance images. *Neuroimage* **23**, S69-84 (2004).

11. Sled, J. G., Zijdenbos, A. P. & Evans, A. C. A nonparametric method for automatic correction of intensity nonuniformity in MRI data. *IEEE transactions on medical imaging* **17**, 87-97 (1998).

12. Fischl, B., Liu, A. & Dale, A. M. Automated manifold surgery: constructing geometrically accurate and topologically correct models of the human cerebral cortex. *IEEE transactions on medical imaging* **20**, 70-80 (2001).

13. Segonne, F., Pacheco, J. & Fischl, B. Geometrically accurate topology-correction of cortical surfaces using nonseparating loops. *IEEE transactions on medical imaging* **26**, 518-529 (2007).

14. Iglesias, J. E., et al. A computational atlas of the hippocampal formation using ex vivo, ultra-high resolution MRI: Application to adaptive segmentation of in vivo MRI. Neuroimage, **115**, 117-137 (2015).

15. Raz, N., et al. Regional brain changes in aging healthy adults: general trends, individual differences and modifiers. *Cereb cortex* **15**, 1676-1689 (2005).

16. Klapwijk, E. T., van de Kamp, F., van der Meulen, M., Peters, S. & Wierenga, L. M. Qoala-T: A supervised-learning tool for quality control of FreeSurfer segmented MRI data. *Neuroimage* **189**, 116-129 (2019).

17. Kaczkurkin, A. N., et al. Neurostructural Heterogeneity in Youths With Internalizing Symptoms. *Biol psychiatry* **87**, 473-482 (2020).
